# *Garcinia mangostana* L. Pericarp Extract and Its Active Compound α-Mangostin as Potential Inhibitors of Immune Checkpoint Programmed Death Ligand-1

**DOI:** 10.3390/molecules28196991

**Published:** 2023-10-09

**Authors:** Sandar Naing, Nichawadee Sandech, Arnatchai Maiuthed, Sumet Chongruchiroj, Jaturong Pratuangdejkul, Pattamapan Lomarat

**Affiliations:** 1 Department of Food Chemistry, Faculty of Pharmacy, Mahidol University, Bangkok 10400, Thailand; sandar.sandarnaing@gmail.com; 2 Centre of Biopharmaceutical Science for Healthy Ageing, Faculty of Pharmacy, Mahidol University, Bangkok 10400, Thailand; nichawadeesandech@gmail.com (N.S.); arnatchai.mai@mahidol.ac.th (A.M.); 3 Department of Pharmacology, Faculty of Pharmacy, Mahidol University, Bangkok 10400, Thailand; 4 Department of Microbiology, Faculty of Pharmacy, Mahidol University, Bangkok 10400, Thailand; sumet.cho@mahidol.ac.th (S.C.); jaturong.pra@mahidol.ac.th (J.P.)

**Keywords:** α-mangostin, xanthones, *Garcinia mangostana* L. pericarp extract, Clusiaceae, PD-L1, chemoprevention, functional ingredient

## Abstract

α-Mangostin, a major xanthone found in mangosteen (*Garcinia mangostana* L., Family Clusiaceae) pericarp, has been shown to exhibit anticancer effects through multiple mechanisms of action. However, its effects on immune checkpoint programmed death ligand-1 (PD-L1) have not been studied. This study investigated the effects of mangosteen pericarp extract and its active compound α-mangostin on PD-L1 by in vitro and in silico analyses. HPLC analysis showed that α-mangostin contained about 30% *w*/*w* of crude ethanol extract of mangosteen pericarp. In vitro experiments in MDA-MB-231 triple-negative breast cancer cells showed that α-mangostin and the ethanol extract significantly inhibit PD-L1 expression when treated for 72 h with 10 µM or 10 µg/mL, respectively, and partially inhibit glycosylation of PD-L1 when compared to untreated controls. In silico analysis revealed that α-mangostin effectively binds inside PD-L1 dimer pockets and that the complex was stable throughout the 100 ns simulation, suggesting that α-mangostin stabilized the dimer form that could potentially lead to degradation of PD-L1. The ADMET prediction showed that α-mangostin is lipophilic and has high plasma protein binding, suggesting its greater distribution to tissues and its ability to penetrate adipose tissue such as breast cancer. These findings suggest that α-mangostin-rich mangosteen pericarp extract could potentially be applied as a functional ingredient for cancer chemoprevention.

## 1. Introduction

The pericarp of mangosteen (*Garcinia mangostana* L.) fruit is a rich source of xanthones, which are bioactive compounds known for their antioxidant, anti-inflammatory, antimicrobial, and anticancer properties [1]. Xanthones are one of the most studied natural compounds for anticancer activity due to their cytotoxic effect. Mangosteen pericarp xanthones, particularly α-mangostin, exhibit potent antioxidant activity, antimicrobial activity [2], and considerable anticancer activity on several cancer cell lines [3]. They have been shown to inhibit several molecular targets in cell signaling cascades involving kinases, cyclooxygenases, and caspases [4]. Moreover, they have been proposed as potential chemopreventive agents for their ability to arrest the cell cycle, suppress tumor cell proliferation, induce apoptosis, and inhibit adhesion, invasion, and metastasis [5]. α-Mangostin (PubChem CID: 5281650; CAS number: 6147-11-1; Molecular Formula: C_24_H_26_O_6_) has been reported to inhibit nuclear factor kappa B (NF-κB) and signal transducer and activator of transcription 3 (STAT3) in animal models and downregulate mitogen-activated protein kinase (MAPK) and protein kinase B (Akt) signaling pathways [6,7]. Likewise, other mangosteen pericarp xanthones such as γ-mangostin, garcinone C, garcinone D, 3-isomangostin, and gartanin also express potential activity on cell signaling pathways in cancer [8,9,10]. Xanthones are mainly present in the pericarp, which is usually discarded for its unpleasant bitter taste. These xanthones could be applied to the production of bioethanol and other valuable products, such as active ingredients for nutraceuticals and functional foods [11]. In addition, a previous study showed the potential applicability of mangosteen pericarp aqueous powder extract as a functional ingredient [12]. Human pilot studies also revealed that mangosteen pericarp extracts are orally bioavailable and have no apparent adverse effects [13]. These findings present the opportunity for the utilization of agricultural waste in the creation of value-added products contributing to a sustainable and circular economy. 

Immune checkpoint proteins programmed death-1 (PD-1) and its ligand programmed death ligand-1 (PD-L1) are mostly expressed on immune cells and tumor cells, respectively. During tumor development, inhibitory immune checkpoint proteins such as PD-L1 are usually overexpressed on cancer cells, causing dysregulation of immune responses which consequently leads to the failure of the immune system to eliminate cancer cells [14,15,16]. PD-L1, also referred to as CD274 or B7-H1, is 33-kDa type 1 transmembrane glycoprotein that contains 290 amino acids with an immunoglobulin-like extracellular region followed by a transmembrane domain and a short intracytoplasmic domain [17]. The structure of PD-L1 is versatile with the potential for dimerization and glycosylated modification [18]. In addition, PD-L1 not only inhibits antitumor immune responses locally, but also enters systemic circulation and interacts with distant cells [19]. The current mAbs therapy is theoretically effective in blocking PD-1/PD-L1 interaction; however, its impact on PD-L1 expression is limited due to poor tumor penetration caused by large molecular size [18]. Therefore, small molecule compounds that can inhibit PD-L1 expression have been proposed to fully stop the biological functions of PD-L1 and effectively inhibit tumor growth. Small molecule inhibitors of PD-L1, such as BMS-202, BMS-1001, and BMS-1166 developed by Bristol-Myers Squibb (BMS), have been shown to block PD-1/PD-L1 interaction by inducing dimerization of PD-L1. X-ray crystal studies revealed that the small molecule induced dimerization of PD-L1, and dimerization in the presence of BMS molecules was confirmed by size-exclusion chromatography and Western blotting using cross-linking agents [20]. Other small molecule inhibitors have been reported to disrupt intracellular pathways and inhibit the expression of PD-L1, as well as induce internalization and degradation of PD-L1 [21,22]. Furthermore, they have advantages such as fewer side effects, a shorter biological half-life, the possibility for self-administration, and being less expensive than mAbs [23]. Overall, immune checkpoint PD-L1 plays a critical role in cancers by allowing cancer cells to escape immune surveillance. Inhibiting PD-L1 expression may be considered as a preventive mechanism against cancer growth and tumorigenesis. Since cancer may exist asymptomatically and be undetected for long periods of time, it is critical to take preventive measures to slow the growth and spread of cancer to other parts of the body. Mangosteen pericarp and its bioactive xanthone α-mangostin are excellent candidates for the study of immune check-point ligand PD-L1 due to their established anticancer activity. Herein, we report the potential activity of mangosteen pericarp extract and its active compound α-mangostin on the expression of PD-L1 and the absorption, distribution, metabolism, excretion, and toxicity (ADMET) parameters of α-mangostin to ensure its efficacy and safety for use as a functional ingredient.

## 2. Results and Discussion

### 2.1. Identification and Quantitation of α-Mangostin by HPLC Analysis

**Figure 1:**
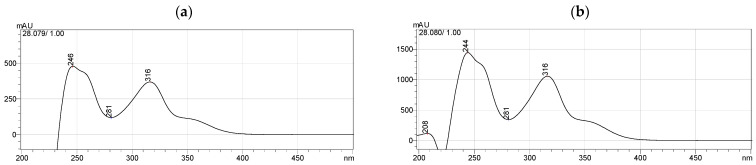
UV spectra of (**a**) the α-mangostin standard and (**b**) its corresponding peak in mangosteen pericarp extract.

**Figure 2:**
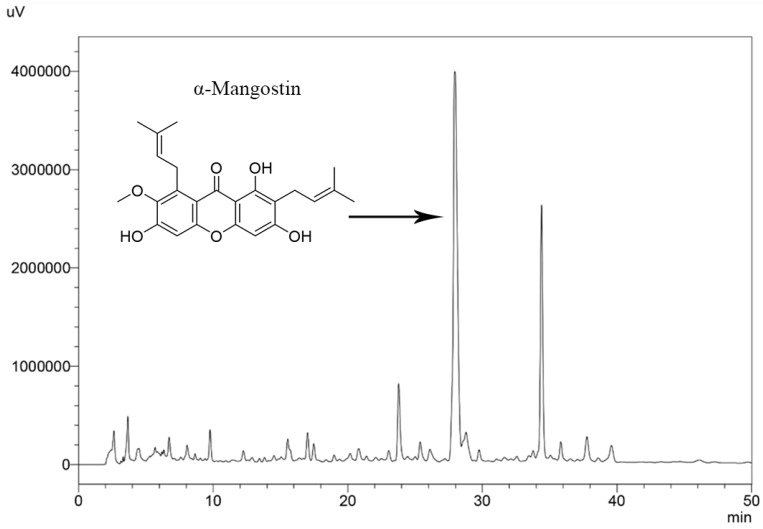
HPLC chromatogram of mangosteen pericarp ethanol extract.

The ethanol extraction of mangosteen pericarp yielded 6.35 g (12.7% *w*/*w*) of yellowish-brown powder. The HPLC method was successfully validated by ICH guideline 2005 [24] and evaluated for specificity, linearity, precision, accuracy, LOD, and LOQ (Table 1). α-Mangostin in the extract was determined by matching its retention time and UV absorption spectrum with those of standard α-mangostin (Figure 1). The amount of α-mangostin in the extract was calculated from the linear regression equation (y = 19587x − 468649) and it was found that the crude ethanol extract of mangosteen pericarp contained 29.4 ± 0.37% *w*/*w* of α-mangostin. The HPLC chromatogram of mangosteen pericarp ethanol extract is shown in Figure 2.

### 2.2. Cancer Cell Line Selection

**Figure 3:**
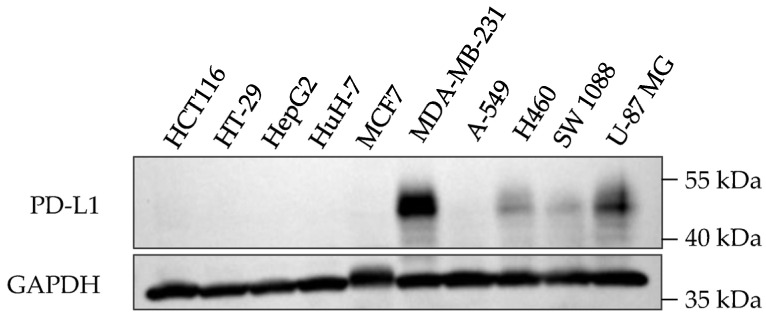
PD-L1 expression in ten cancer cell lines.

PD-L1 proteins undergo post-translational protein modification including glycosylation, and the glycosylated form of PD-L1 (~50 kDa) is the most detected form in cancer cells [25]. The expression of PD-L1 on cancer cell surfaces varies due to diverse mechanisms, including tumor type, genetic factors, immune responses, and interactions with T cells [26]. Breast cancer cell line MDA-MB-231 was reported to have higher levels of PD-L1 expression than other tumor cell lines, such as HepG2, A-549, and A375 [26]. In the present study, the glycosylated form of PD-L1 was detected in four out of the ten cancer cell lines investigated, which were MDA-MB-231, H460, SW 1088, and U-87 MG (Figure 3). Among these PD-L1 expressed cells, MDA-MB-231 triple-negative breast cancer cells consistently showed the highest levels of the glycosylated form of PD-L1 expression compared to other cell lines. Hence, this cell line was selected for further analysis.

### 2.3. Cell Viability

**Figure 4:**
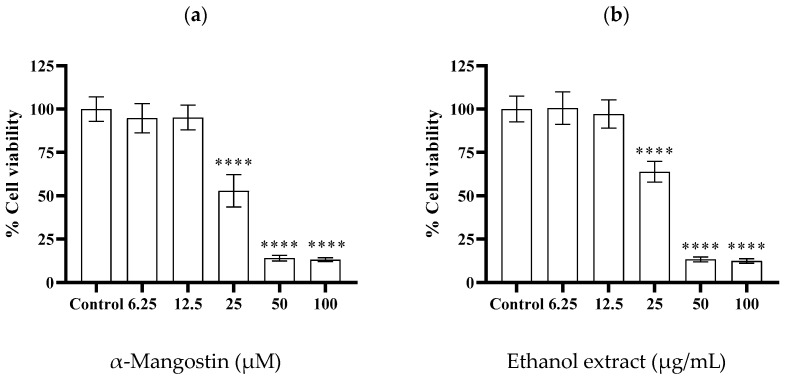
Cell viability of MDA-MB-231 triple-negative breast cancer cells after 24 h treatment with various concentrations of (**a**) α-mangostin (6.25, 12.5, 25, 50, and 100 µM), and (**b**) mangosteen pericarp ethanol extract (6.25, 12.5, 25, 50, and 100 µg/mL). Data are represented as means ± SDs (*n* = 3). Statistics: one way ANOVA with (****) *p* < 0.0001 versus control group.

The viability of cells treated with α-mangostin and mangosteen pericarp ethanol extract was examined via an MTT assay. The concentrations of α-mangostin and mangosteen pericarp ethanol extract that showed cell viability at 50% (IC_50_) were found to be ~23 µM and ~26 µg/mL, respectively (Figure 4a,b).

### 2.4. Western Blotting

**Figure 5:**
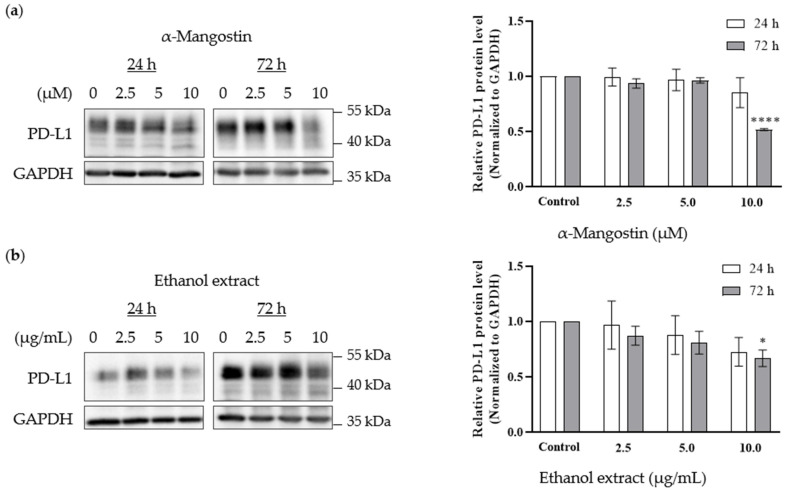
Effects of α-mangostin and mangosteen pericarp ethanol extract on PD-L1 expression in MDA-MB-231 triple-negative breast cancer cells. Western blot analysis demonstrates a decrease in PD-L1 expression levels following 24 h and 72 h treatment with (**a**) α-mangostin (2.5, 5, 10 µM) (*n* = 3) and (**b**) mangosteen pericarp ethanol extract (2.5, 5, 10 µg/mL) (*n* = 5). Data are represented as means ± SEMs of PD-L1 intensity normalized to GAPDH intensity. Statistics: one way ANOVA with (*) *p* < 0.05 and (****) *p* < 0.0001 versus control group.

The effects of α-mangostin and mangosteen pericarp ethanol extract on PD-L1 expression in the MDA-MB-231 triple-negative breast cancer cell line was investigated by Western blot analysis. The treatment doses were determined based on the MTT assay results. Non-toxic concentrations of α-mangostin (2.5, 5, and 10 µM) and non-toxic concentrations of ethanol extract (2.5, 5, and 10 µg/mL) were selected. As shown in Figure 5a,b, α-mangostin and the ethanol extract reduced the expression of PD-L1 in MDA-MB-231 cells at doses of 10 µM and 10 µg/mL, respectively. Both tested samples exhibited a significant decrease in PD-L1 expression after 72 h treatment, suggesting that xanthone compounds need longer time to exert their activity on PD-L1 expression in MDA-MB-231 cancer cells. The inhibition of PD-L1 expression by the ethanol extract is likely due to α-mangostin, as the ethanol extract contained ~30% α-mangostin according to HPLC analysis.

PD-L1 is heavily glycosylated, and it was reported that inhibition of glycosylation has been associated with enhanced anti-PD-1/PD-L1 therapy, as well as degradation of the immunosuppressive function of PD-L1 [25]. Glycosylation also maintains the stability of PD-L1, and inhibition of PD-L1 glycosylation is considered to be a potential mechanism for immune checkpoint therapy [27]. In this study, PD-L1 bands were detected at ~50 kDa, indicating the highly glycosylated form of PD-L1. The Western blot analysis revealed that the lower molecular forms of PD-L1 were detected at ~40 kDa in the treatment groups, suggesting that xanthones may likely partially inhibit PD-L1 glycosylation and/or change the constitutive expression pattern of PD-L1 in MDA-MB-231 triple-negative breast cancer cells.

### 2.5. Immunofluorescence Staining

**Figure 6:**
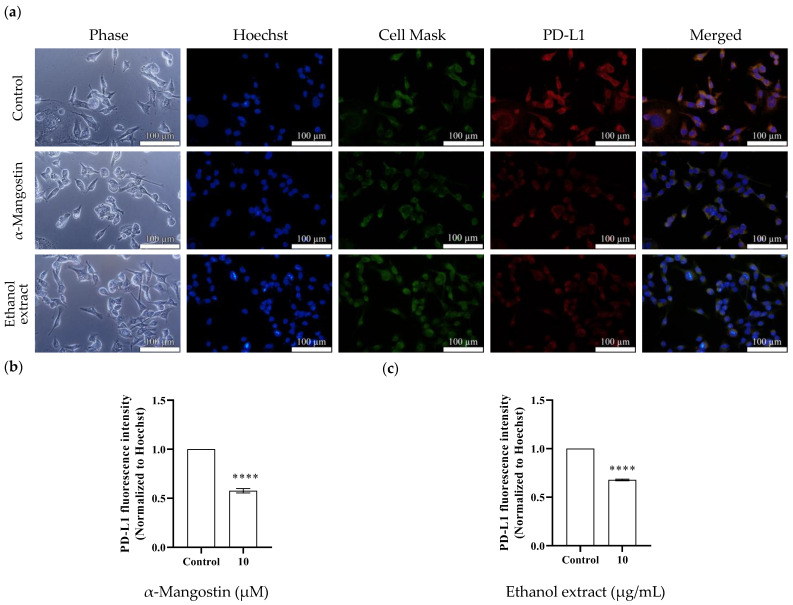
Effects of α-mangostin and mangosteen pericarp ethanol extract on PD-L1 expression in MDA-MB-231 triple-negative breast cancer cells. (**a**) Immunofluorescence staining for PD-L1 expression in the control group vs. the α-mangostin-treated group vs. the ethanol extract-treated group. Cell nuclei were stained with Hoechst (blue), plasma membranes were stained with Cell Mask^TM^ (green), and PD-L1 proteins were stained with Alexa Fluor^®^ 594 conjugate (red). The bar charts represent the immunofluorescence intensity of PD-L1 in (**b**) the α-mangostin-treated group and (**c**) the ethanol extract-treated group. Data are represented as means ± SEMs (*n* = 3) and were normalized with Hoechst signals before comparisons between groups. Statistics: one way ANOVA with (****) *p* < 0.0001.

Immunofluorescence staining was performed to examine the influence of α-mangostin and mangosteen pericarp extract on PD-L1 expression in MDA-MB-231 cancer cells. In accordance with the Western blot results, α-mangostin and ethanol extract treatments remarkably reduced the fluorescence signal of PD-L1 (Figure 6). These results indicate that α-mangostin and the ethanol extract of mangosteen pericarp show potential cancer chemopreventive activity by inhibiting the immune checkpoint ligand PD-L1.

### 2.6. ADMET Prediction

The physicochemical properties of α-mangostin were analyzed using Lipinski’s rule of five parameters to estimate oral bioavailability. The Lipinski’s rule of five comprises four parameters, which are a molecular weight of ≤500 Da, a number of hydrogen bond donors ≤5, a number of hydrogen bond acceptors ≤10, and an octanol–water partition coefficient of LogP ≤ 5 [28]. LogP of greater than 5 is usually considered highly lipophilic and contributes to low solubility, poor oral absorption, and a high risk of toxicity due to the tendency to bind to undesired targets [29]. 

For oral drugs, LogP greater than 1 or less than 4 is generally considered to indicate optimal physicochemical and ADME properties [29]. The ADMET descriptor model was applied with the Atom-based LogP (ALogP) and molecular polar surface area (PSA) to predict the cell wall permeability of the compound. Cell wall permeability is important for proper intestinal absorption and penetration across the blood–brain barrier. Previous studies have reported that the upper limit of PSA and ALogP98 cutoff values with a 95% confidence level are 131.6 Å^2^ and 5.88, respectively [30]. According to the model, compounds with a molecular weight between 400 and 500 Da that follow PSA-ALogP98 criteria have acceptable physicochemical properties for orally delivered compounds.

As shown in Table 2, α-mangostin showed a high ALogP value of 5.935, which is greater than the cutoff value of 5.88, indicating the lipophilic nature of the compound. High lipophilicity contributes to high cell permeability, the greater distribution of drugs to tissues and tends to increase plasma protein binding, which could limit the availability of the free drug for distribution throughout the body [31]. However, the lipophilic nature of α-mangostin could be advantageous in certain cancers which occur in fat tissue, such as breast cancer. This is because the lipophilic compound may have greater potential to penetrate fat tissue and reach tumor microenvironments in these fat tissues.

The in vivo animal studies and randomized human trial studies showed that α-mangostin is orally bioavailable, especially when provided using an oily vehicle. A human study reported the detection of metabolites of α-mangostin in plasma and in urine; however, approximately 2% of the ingested dose was absorbed [13]. These studies indicated the low absorption of α-mangostin in the body, which can be attributed to its high LogP value and lipophilic nature. The ADMET prediction result (Table 3) showed low water solubility and intestinal absorption of α-mangostin, suggesting poor oral bioavailability. The reported experimental solubility property of α-mangostin is 0.000203 mg/L according to the PubChem database [32], indicating low water solubility which agrees with the predicted result.

α-Mangostin is predicted to be non-mutagenic, though it is hepatotoxic and has carcinogenic potential in male rats according to the US National Toxicology Program (NTP) model. ADMET prediction and TOPKAT analysis of α-mangostin are shown in Table 3 and Table 4. Highly lipophilic compounds tend to have high plasma protein binding. The predicted result showed that α-mangostin has high ADMET plasma protein binding of more than 90%. This high tendency to bind to plasma protein may affect the distribution and metabolism of α-mangostin in the body. It is likely that the high plasma protein binding property of α-mangostin contributes to prolonged detection of up to 24 h in animal studies [33]. Moreover, this binding may result in reduced biological activity due to unavailability of the unbound drug limiting its ability to exert biological activity. The predicted rat oral LD_50_ of α-mangostin was estimated to be 0.168 g/kg (equivalent to 168 mg/kg). The reported LC_50_ of α-mangostin and mangosteen extract was 150 and 231 mg/kg, respectively, when given via the intraperitoneal route in mice [34]. Although the prediction result provides an estimation of the oral LD_50_ dosage, it should be noted that the predicted dosage may not be aligned with experimental results and further validation is necessary.

### 2.7. Molecular Docking

To further analyze the molecular mechanism of α-mangostin, a molecular docking procedure was performed. α-Mangostin was docked on the PD-L1 dimer (PDB ID: 5N2F) and the binding energy and binding conformation of α-mangostin on the PD-L1 dimer pocket was studied. The docking procedure was validated by performing redocking of the native ligand into its receptor. Docking software is considered reliable if it generates a root-mean-square deviation (RMSD) value of less than 1.5 or 2 Å between the native ligand and redocked ligand [35]. 

**Figure 7:**
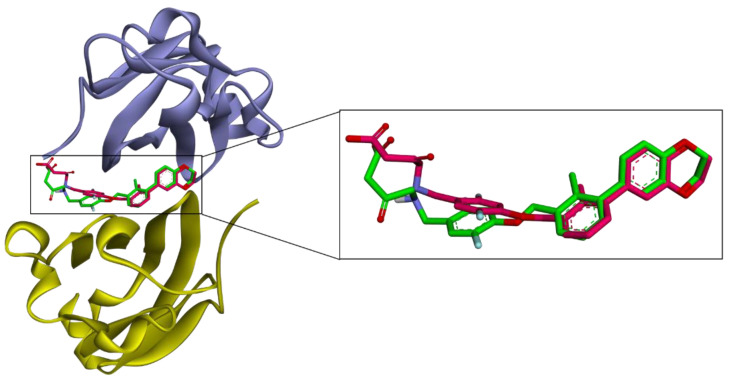
Validation of molecular docking. Superposition of the AutoDock 4.2.6 generated pose (pink) and the native ligand (green) of the PD-L1 dimer (PDB ID: 5N2F). The RMSD value between the native ligand and generated ligand was 1.261 Å. Chain A is purple and chain B is yellow.

To obtain unbiased results, the coordinates and conformations of native ligands were randomized before redocking. As shown in Figure 7, the RMSD value between the co-crystallized ligand and redocked ligand was 1.261 Å and the docking procedure was validated.

The crystal structure of PDB ID: 5N2F with small molecule BMS-200 revealed that the binding of small molecules induces dimerization of PD-L1 protein and the formation of a 16 Å long cylindrical hydrophobic pocket. The pocket is closed from one side by π–σ interaction with _A_Tyr56, while the other side is strongly stabilized by π–π stacking interaction with _B_Tyr56 with halogen bonding between the fluorine atom with a minor contribution from _A_Asp122. The center cleft of the PD-L1 dimer is stabilized by hydrophobic π–alkyl interactions with _AB_Met115 and by additional contacts including hydrogen bonding with _A_Ala121 and alkyl interaction with _A_Tyr123. Additionally, BMS-200 formed two hydrogen bonds with _A_Thr20 and _B_Gln66 [20]. _A_Tyr56 has been shown to be a key residue for ligand binding and its orientation characterized the conformation of the PD-L1 dimer into open and closed states [36]. Based on the molecular modeling studies on 29 BMS inhibitors, the three common residues that play a critical role in ligand binding to PD-L1 protein are found to be Tyr56, Asp122, and Lys124 [37]. 

**Figure 8:**
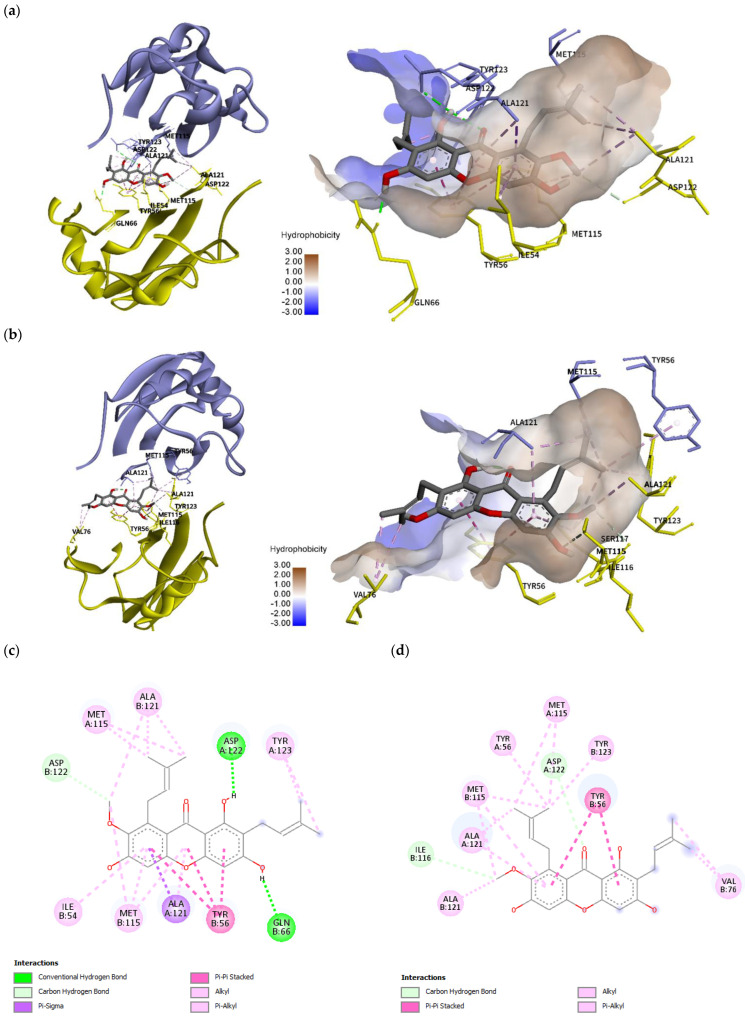

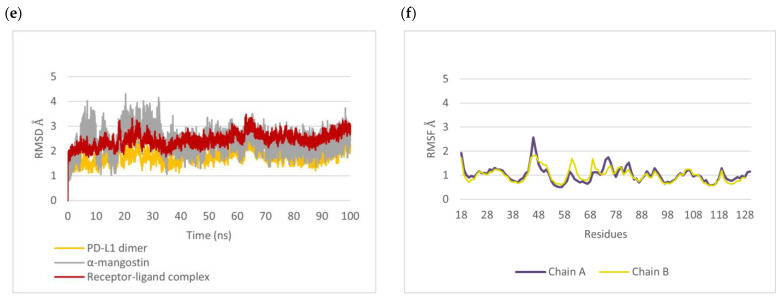
Molecular docking and molecular dynamics simulation of α-mangostin with the PD-L1 dimer (PDB ID: 5N2F), with chain A in purple and chain B in yellow. Binding conformations of α-mangostin inside the PD-L1 dimer pocket (**a**) before simulation with a docking energy of −10.86 kcal/mol and (**b**) after 100 ns simulation. Binding free energy was –16.2656 kcal/mol. Two-dimensional diagrams of α-mangostin interacting with the PD-L1 dimer are shown (**c**) before simulation and (**d**) after 100 ns simulation. (**e**) Root mean square deviation (RMSD) of α-mangostin, the PD-L1 dimer (residue 18 to 130 of chain A and chain B), and the α-mangostin–PD-L1 dimer complex. (**f**) Root mean square fluctuation (RMSF) of Cα atoms of residue 18 to 130 from chain A and chain B of the PD-L1 dimer.

In comparison to the BMS-200 molecule, α-mangostin does not possess a sufficiently long molecular structure to completely fill in the 16 Å long cylindrical hydrophobic pocket of PD-L1. In the crystal structure, _A_Tyr56 is moved backwards because the ligand binding creates a tunnel. Binding mode analysis using the BIOVIA Discovery Studio Visualizer (version 21.1.0.20298, San Diego, Dassault Systèmes) revealed that α-mangostin was able to interact with _AB_Tyr56 at a low binding energy of -10.8 kcal/mol; however, the interacting residues were fewer, and the conformation did not generate any hydrogen bonds. In its best binding conformation (Figure 8a,c), α-mangostin did not interact with _A_Tyr56. However, it closed the tunnel by π–alkyl/alkyl interaction with the residues of _A_Met115 and _B_Ala121. The methoxy group of α-mangostin also provided carbon hydrogen bonds with _B_Ile116 and _B_Asp122. The xanthone core is stabilized by π–σ interaction with _A_Ala121 supported by _B_Met115 through π–alkyl/alkyl interaction. _B_Tyr56 generated π–π stacking interaction stabilizing the central part. The other side chain of α-mangostin interacted with _A_Tyr123 through π–σ and π–alkyl/alkyl interaction. The two hydroxy groups of α-mangostin formed hydrogen bonds with _A_Asp122 and _B_Gln66. Additionally, binding with _A_Tyr123, _B_Ile54, and _B_Gln66 stabilized α-mangostin inside the tunnel.

### 2.8. Molecular Dynamics Simulation

The binding mode and binding stability of α-mangostin were further analyzed by conducting molecular dynamics (MD) simulations. As shown in Figure 8b,d, the binding conformation after 100 ns simulation revealed that α-mangostin interacted with the _AB_Tyr56 of PD-L1 with additional interactions including _AB_Met115, _AB_Ala121, _B_Val76, _B_Ile116, _A_Asp122, and _B_Tyr123. The result suggested that the high flexibility of _A_Tyr56 allowed α-mangostin to interact with both forms of Tyr56 after 100 ns simulation. Molecular dynamics study of the natural bioactive compounds capsaicin, zucapsaicin, 6-gingerol, and curcumin with the PD-L1 dimer revealed that the key residues Ile54, Tyr56, Met115, and Ala121 play a role in stabilizing protein–ligand complexes [38]. This study also revealed that Tyr56, Met115, and Ala121 were key residues for ligand binding. Additionally, the high flexibility of _A_Tyr56 supports ligand binding and the stability of α-mangostin inside the binding tunnel of PD-L1. Out of 10 interacting residues, α-mangostin interacted with more binding residues on chain B of PD-L1.

The RMSD trajectory (Figure 8e) showed that α-mangostin fluctuated by around ~3 Å in the first 35 ns; however, it eventually regained stability thereafter and was typically stable inside the PD-L1 dimer pocket throughout the 100 ns simulation. The RMSF of alpha carbon atoms was also calculated to identify the fluctuating area of PD-L1 dimers. The IgV-like domain (amino acids 19–127) of PD-L1, which represents the target site for mAbs, peptides, and small molecules, was found to be more stable (<1.5 Å RMSF) than that in the IgC-like domain (amino acids 128 to 239). As shown in Figure 8f, the residues in the loop region of the IgV domain, particularly the BC loop (amino acids 44 to 48) of chain A, showed high RMSF values of ~2 Å. The previous molecular simulation study of food-derived polyphenols also revealed a high fluctuation of ~5 Å in the BC loop of the PD-L1 dimer [39]. This study showed more stability for the BC loop of the PD-L1 dimer. Moreover, the residues on chain B showed more stability than those on chain A. The molecular dynamics results indicate that the target site on the PD-L1 dimer is likely to support the binding of α-mangostin, and the complex showed conformational stability throughout the 100 ns simulation. 

### 2.9. Binding Free Energy Calculation

The binding free energies (ΔG_bind_) of the last 10 ns of stable MD trajectories were calculated using the MMPBSA approach. The binding free energy and the contributions of the components of the system are summarized in Table 5. The free binding energy of the PD-L1 dimer with α-mangostin was −16.2656 kcal/mol. The result shows that gas phase molar mechanics (ΔE_gas_), non-polar binding free energy (ΔG_non-polar_), and van der Waals (ΔE_vdW_) energy represent the main driving forces of binding with the PD-L1 dimer. The contributions of non-polar energy indicate the hydrophobic interaction of the compound inside the binding site. This result further verifies that α-mangostin has the potential to stabilize the dimer form of PD-L1, which may lead to the degradation of PD-L1. Further studies are needed to explore the potential activity of α-mangostin on PD-L1 dimerization, as well as its inhibitory effect on PD-1/PD-L1 interaction.

## 3. Materials and Methods

### 3.1. Plant Materials

Mangosteen fruits were collected from Chanthaburi Province, Thailand. Their authenticity (Voucher specimen No. WGM0615) was confirmed by Assoc. Prof. Dr. Omboon Vallisuta, Department of Pharmacognosy, Mahidol University, Thailand. Mangosteen pericarp powder and purified α-mangostin standard [40] were provided by Miss Jutima Samer, Department of Physiology, Faculty of Pharmacy, Mahidol University.

### 3.2. Chemicals and Reagents

LCMS grade ethanol, methanol, acetic acid, and acetonitrile were purchased from Thermo Fisher Scientific, Waltham, MA, USA. Dulbecco’s modified Eagles’s medium (DMEM), Roswell Park Memorial Institute (RPMI) 1640 supplemented with 10% FBS, 1% L-glutamine, 1% penicillin/streptomycin, and 1% HEPES buffer solution were purchased from Gibco^TM^, Thermo Fisher Scientific, Waltham, MA, USA. Dimethyl sulfoxide (DMSO), Trypsin-EDTA, 3-(4,5-dimethylthiazol-2-yl)-2,5-diphenyl-2H-tetrazolium bromide (MTT), BCA protein analysis kits, Hoechst 33342, and Cell Mask™ Green Plasma membrane stain were also purchased from Thermo Fisher Scientific, Waltham, MA, USA.

### 3.3. Extraction of Mangosteen Pericarp

The mangosteen pericarp extract was prepared by the previously described method [41]. Briefly, the fresh pericarps were separated from mangosteen fruits and cleaned thoroughly. They were cut into pieces and dried in a hot air oven at 60 °C (Memmert GmbH + Co. KG, Schwabach, Germany). They were then crushed into powder and stored at −20 °C. Fifty grams of mangosteen pericarp powder was macerated with 250 mL of absolute ethanol and kept in the shaker incubator (New Brunswick Scientific, CT, USA) at 60 °C at 80 rpm for 24 h. The supernatant was filtered through Whatman No.1 filter paper and the filtrate was concentrated using a rotary evaporator (Buchi, Flawil, Switzerland). The marc was re-extracted twice by the same procedure. The concentrated ethanol extract was freeze-dried (Martin Christ Gefriertrocknungsanlagen GmbH, Osterode am Harz, Germany) and stored at −20 °C.

### 3.4. Identification and Quantification of α-Mangostin by HPLC Analysis

#### 3.4.1. Sample Preparation

α-Mangostin standard was prepared at a 2500 µg/mL concentration by dissolving it in methanol. The stock solutions were further diluted to obtain 30, 100, 500, and 1000 µg/mL concentrations. For sample preparation, 15 mg of mangosteen pericarp extract was accurately weighed and dissolved in 1 mL of methanol in a volumetric flask. All samples and standard solutions were filtered through 0.22 µm syringe filters before being injected into the HPLC system.

#### 3.4.2. HPLC Analysis of α-Mangostin in Mangosteen Pericarp Extract

The mangosteen pericarp extract was analyzed using a Shimadzu Nexera HPLC system (Shimadzu Scientific Instruments, Inc., Columbia, MD, USA) with a column oven equipped with a DGU-405 degassing unit, LC-40D XR pumps, an SIL-40C autosampler, an SPD-M40 photodiode array detector, and LabSolutions software version 5.111. The HPLC analysis was carried out on a Purospher^®^ STAR RP-18 endcapped column (150 mm × 4.6 mm, particle size 5 µm, Merck KGaA, Darmstadt, Germany) with a Purospher^®^ STAR RP-18 endcapped (particle size 5 µm) LiChroCART^®^ 4-4 (Merck KGaA, Darmstadt, Germany) guard column. The HPLC analytical method was developed by modifying a previously described method [42] and was validated according to ICH guidelines [24]. The method was validated for linearity, precision, accuracy, limit of detection (LOD), and limit of quantitation (LOQ). The mobile phase used was 2% acetic acid in water (A) and acetonitrile (B). The gradient program was as follows: 0–2 min 30–50% B, 2–21 min 50–80% B, 21–23 min 80% B, 23–30 min 80–95% B, 30–50 min 95% B, which was performed at a column temperature of 25 °C. The flow rate was 0.6 mL/min, and the injection volume was 10 µL. The total separation time was 50 min and was monitored at 281 nm. α-Mangostin was identified and quantified using the external standard method. The retention time and the UV spectrum of the α-mangostin standard were compared with those of the corresponding peak in the extract.

### 3.5. Cell Culture and Cancer Cell Line Selection

Cancer cell lines consisting of two colon cancer cell lines (HCT116 (Lot number #70040763, ATCC) and HT-29 (Lot number #70019050, ATCC)), two liver cancer cell lines (HepG2 (Lot number #70039681, ATCC) and HuH-7 (JCRB0403, JCRB Cell Bank)), two breast cancer cell lines (MCF7 (Lot number #70033778, ATCC) and MDA-MB-231 (Lot number #70029549, ATCC)), two lung cancer cell lines (A-549 (Lot number #70035208, ATCC) and H460 (Lot number #70039818, ATCC)), and two brain cancer cell lines (SW 1088 (Lot number #70036445, ATCC) and U-87 MG (Lot number #70029548, ATCC)) were examined for their PD-L1 expression levels. Cells were cultivated in a suitable medium, such as low- or high-glucose DMEM or RPMI. Cells were maintained at 37 °C in a humidified atmosphere containing 5% CO_2_. The culture medium was changed every two days. Cells were trypsinized using 0.5% trypsin–EDTA when they reached 80% confluence, and their PD-L1 expression was determined using the Western blot method. Cancer cell lines with high PD-L1 expression levels were selected for further experiments.

### 3.6. Cell Treatment

The cancer cell line with high PD-L1 expression was treated with α-mangostin or mangosteen pericarp extract for 24 h or 72 h, and the concentration range was 6.25, 12.5, 25, 50, and 100 µM or µg/mL for the cell viability assay and 2.5, 5, and 10 µM or µg/mL for Western blot analysis. The extract or α-mangostin was dissolved in DMSO and diluted with culture medium so that the final concentration of DMSO was less than 1% *v*/*v*. The control group was treated with 1% *v*/*v* DMSO.

### 3.7. Cell Viability

Approximately 1 × 10^4^ cells/well of the cancer cell line with high expression of PD-L1 were seeded in 96-well plates and cultured for 24 h. They were treated with different concentrations of α-mangostin (6.25, 12.5, 25, 50, and 100 µM) or the mangosteen pericarp extract (6.25, 12.5, 25, 50, and 100 µg/mL) for 24 h. The control group was treated with 1% *v*/*v* DMSO. After treatment, 100 µL of MTT solution (0.5 mg/mL) was added to each well, and cells were incubated at 37 °C for 3 h. After incubation, the MTT solution was discarded and 100 µL of DMSO was added to dissolve formazan crystals. The plate was gently agitated until the formazan precipitates were dissolved. The absorbance was measured at 570 nm through the use of a microplate reader (Agilent BioTek, CA, USA) to determine cell viability. The experiment was conducted in triplicate and cell viability was calculated by the following equation.

$$\%\;\mathrm{Cell}\;\mathrm{viability}=\frac{\mathrm{Absorbance}\;\mathrm{of}\;\mathrm{treated}}{\mathrm{Absorbance}\;\mathrm{of}\;\mathrm{control}}\;\times \;100$$

### 3.8. In Vitro Analysis of the Effect of α-Mangostin and Mangosteen Pericarp Extract on PD-L1 Expression

#### 3.8.1. Western Blotting

Cancer cells at a concentration of 3 × 10^4^ for 72 h treatment and 3 × 10^6^ cells/well for 24 h treatment were seeded in 6-well plates and cultured for 24 h. They were treated with α-mangostin or mangosteen pericarp extract with concentration ranges of 2.5, 5, and 10 µM or µg/mL, respectively. Cells were lysed in a 1X RIPA lysis buffer cocktail containing 1% protease inhibitor and 1% phenylmethyl sulfonyl fluoride (PMSF) and then sonicated using a sonication probe (Vibra-Cell, Sonics, CT, USA) at 30% amplitude on ice for 10 s. The lysed cells were centrifuged at 12,500 rpm at 4 °C for 15 min and the supernatants were collected. Protein concentration was determined using a BCA protein analysis kit (Thermo Fisher Scientific, Waltham, MA, USA). Proteins (30 μg) were loaded onto 10% sodium dodecyl sulfate–polyacrylamide gel electrophoresis (SDS-PAGE). The separated proteins were transferred onto polyvinylidene difluoride (PVDF) membranes via the gel sandwich technique. The transferred membrane was blocked with 5% skim milk for 2 h at room temperature followed by incubation with primary antibodies, PD-L1 (E1L3N^®^ XP^®^ Rabbit mAb #13684, Cell Signaling Technology, Beverly, MA, USA) at 1:2000 dilution and GAPDH (D16H11 XP^®^ Rabbit mAb (HRP Conjugate) #8884, Cell Signaling Technology, MA, USA) at 1:10000 dilution in 5% BSA (Sigma-Aldrich, St. Louis, MO, USA) at 4 °C overnight. Secondary antibodies used were horseradish peroxidase (HRP)-conjugated anti-rabbit IgG (#7074, Cell Signaling Technology, MA, USA) and anti-biotin (#7075, Cell Signaling Technology, MA, USA) at 1:2000 dilution. The protein bands were exposed using enhanced chemiluminescence reagents and detected by gel imaging and a documentation system (iBright 1500, Thermo Fisher Scientific, Waltham, MA, USA). The signal intensities were calculated using iBright analysis software (version 5.1.0) and all protein expression levels were normalized to the expression level of GAPDH.

#### 3.8.2. Immunofluorescence Staining

Cells were seeded in 8-well chamber slides (µ-Slide 8 well, ibidi GmbH, Gräfelfing, Germany) at a density of 1 × 10^4^ and cultivated for 24 h. They were treated with 10 µM of α-mangostin or 10 µg/mL of extract for 72 h. After treatment, cells were fixed with 4% formaldehyde for 30 min at room temperature and then blocked with 5% BSA containing 0.1% Triton-X100 (Merck, Rahway, NJ, USA) for 1 h at room temperature. Incubation with the PD-L1 antibody at 1:200 dilution was performed at 4 °C overnight. Cells were washed three times with PBS and incubated with fluorescence secondary antibody Alexa Fluor^®^ 594 Conjugate (#8889, Cell Signaling Technology, MA, USA) at 1:500 dilution for 2 h at room temperature in the dark. Cells were counterstained with Hoechst for visualization of nuclei and Cell Mask™ (1:500) for visualization of cell membranes. Images were captured using an Olympus fluorescence microscope (IX83, Evident Life Science, Waltham, MA, USA) equipped with CellSens imaging software, Olympus CellSens dimension 3.1.1 (Build 21264). The maximum emission and excitation wavelengths of Alexa Fluor^®^ 594, Hoechst, and Cell Mask^TM^ were 590–617, 361–497, and 522–535 nm, respectively. The fluorescence intensities of images were quantified by ImageJ software (version 1.54d) [43]. 

### 3.9. ADMET Prediction of α-Mangostin

The absorption, distribution, metabolism, excretion, and toxicity (ADMET) potential of α-mangostin was predicted using BIOVIA Discovery Studio 2021 Client, version 21.1.0.20298 (Dassault Systèmes Biovia Corp, San Diego, CA, USA). Pharmacokinetic parameters including aqueous solubility, human intestinal absorption, blood–brain barrier penetration, plasma protein binding, cytochrome P450 2D6 inhibition, and hepatotoxicity potential were predicted using ADMET descriptors. The toxicity potential of mangosteen pericarp xanthones, such as Ames mutagenicity, carcinogenicity, skin irritation, ocular irritation, and aerobic biodegradability, was predicted using the TOPKAT predictive toxicology module. 

### 3.10. In Silico Analysis of the Inhibitory Activity of α-Mangostin on PD-L1 Protein

#### 3.10.1. Molecular Docking

The molecular docking procedure was carried out using AutoDock 4.2.6 [44]. The crystal structure of human PD-L1 complexed with the small molecule inhibitor BMS-200 (PDB ID: 5N2F) [20] with a resolution of 1.7Å was downloaded from the RCSB protein data bank (http://www.rcsb.org/, accessed on 3 December 2021). The crystal structure was prepared for docking using AutoDock tools. Briefly, water, ligand, and hetero atoms were deleted from the crystal structure and missing atoms were repaired. Then, polar hydrogens and Gasteiger partial charges were added for electrostatic interactions. The 3D structure of α-mangostin was downloaded from the PubChem database [32]. The binding pocket was centered on the native ligand and the coordinates were as follows: x = 32.391, y = 12.721, z = 133.816. The grid box was set at dimensions of 40 Å × 40 Å × 40 Å with 0.375 Å spacing. The Lamarckian genetic algorithm was applied, and 100 docking runs were carried out. All other parameters were set at default values. The conformation with the best binding affinity was selected for molecular dynamics simulation. 

#### 3.10.2. Molecular Dynamics Simulation

NAMD software version 2.14 (downloaded from http://www.ks.uiuc.edu/Research/namd/, accessed on 15 January 2022) [45] was used to run molecular dynamics simulation of the PD-L1 and α-mangostin complex. The protein structure file and parameter files were generated using the CHARMM-GUI input generator [46]. The complex was solvated in the water box with a minimum padding of 20 Å × 20 Å × 20 Å, and the system was neutralized with an ionic strength of NaCl to 0.15 mol/L using VMD software version 1.9.4 [47]. The simulation was initiated with an energy minimization step, followed by heating and equilibrating the system. Energy minimization was performed for 20,000 steps, and the system was then heated for 10,000 steps by gradually increasing the temperature from 0 to 310 K. Afterward, the system was equilibrated for 200,000 steps by applying normal temperature and pressure at 310 K and 1 atm as controlled by the Nosé–Hoover Langevin piston method. Then, the production run of 100 ns (50,000,000 steps) was carried out using the same conditions as in the equilibration step. The trajectories were recorded every 2 ps for analysis. Followed by molecular simulation, the RMSD and RMSF were calculated to analyze the stability of the complex.

#### 3.10.3. Binding Free Energy Calculation

The trajectory acquired from molecular dynamics simulation was subjected to calculation of the binding free energy using the MMPBSA method computed with the CaFE (Calculation of Free Energy) tool [48]. A total of 1000 frames extracted from the last 10 ns of the trajectory file were subjected to binding free energy calculation. According to the MMPBSA method computed by CaFE, the binding free energy (${\Delta G}_{\mathrm{bind}}$) is calculated as follows.

$${∆G}_{\mathrm{bind}}=∆H\;-T∆S$$

$$∆H={∆E}_{\mathrm{gas}}+{∆G}_{\mathrm{sol}}^{\mathrm{polar}}+{∆G}_{\mathrm{sol}}^{\mathrm{nonpolar}}$$

However, due to high computational cost and the inaccuracy of current methods for entropy calculation, T∆S is ignored in CaFE. Therefore, the final equation is as follows.

$${∆G}_{\mathrm{bind}}={∆E}_{\mathrm{gas}}+{∆G}_{\mathrm{sol}}^{\mathrm{polar}}+{∆G}_{\mathrm{sol}}^{\mathrm{nonpolar}}$$

$${∆G}_{\mathrm{bind}}=\mathrm{Binding}\;\mathrm{free}\;\mathrm{energy}$$

∆H=Enthalpy change

T = Temperature in Kelvin

∆S=Entropy change

$${\Delta E}_{\mathrm{gas}}=\mathrm{Electrostatic}\;\mathrm{energy}$$

$${∆G}_{\mathrm{sol}}^{\mathrm{polar}}=\mathrm{Polar}\;\mathrm{solvation}\;\mathrm{energy}$$

$${∆G}_{\mathrm{sol}}^{\mathrm{nonpolar}}=\mathrm{Nonpolar}\;\mathrm{solvation}\;\mathrm{energy}$$

### 3.11. Statistical Analysis

Data were expressed as the mean ± standard deviation (SD) or the mean ± standard error of mean (SEM) of at least three triplicate experiments. Statistical significance of the mean values between control and treatment groups was analyzed using one way ANOVA in GraphPad Prism 8 software (version 8.0.1) (San Diego, CA, USA). *p* < 0.05 was considered statistically significant. 

## 4. Conclusions

This is the first report of the potential inhibitory activity of mangosteen pericarp extract and its active compound α-mangostin on the immune checkpoint ligand PD-L1. Through in vitro and in silico analyses, this study uncovered the potential activity of α-mangostin on the immune checkpoint ligand PD-L1. Our findings revealed that mangosteen pericarp ethanol extract and its active compound α-mangostin possess cytotoxic activity and potentially inhibit PD-L1 expression, as well as partially inhibit the glycosylation of PD-L1 in the MDA-MB-231 triple-negative breast cancer cell line. Furthermore, α-mangostin may likely dimerize PD-L1 and may potentially lead to the degradation of PD-L1 while also potentially inhibiting PD-1/PD-L1 interactions. Further research is needed to verify the activity of α-mangostin in other cancer cell lines and in animal models. In addition, the lipophilic nature of α-mangostin may enhance tumor cellular penetration. For application in nutraceuticals and functional foods, the solubility of α-mangostin should be addressed to enhance the oral absorption and bioavailability of α-mangostin. Our study suggests that α-mangostin-rich mangosteen pericarp extract has the potential to be developed for nutraceuticals and functional foods that may provide cancer chemoprevention benefits.

## Figures and Tables

**Table 1:** HPLC method validation.

Parameters	Results
Linearity range (R^2^ > 0.99)	0.9993
Accuracy (recovery 80–120%)	90–101%
Precision (%RSD ≤ 2)	≤0.72
Limit of detection (LOD)	0.23 µg/mL
Limit of quantitation (LOQ)	0.7 µg/mL

**Table 2:** Lipinski’s parameters of α-mangostin.

Parameters	Results
Molecular weight (≤500 Da)	410.46 Da
ALogP (≤5.88)	5.935
Number of hydrogen acceptors (≤10)	6
Number of hydrogen donors (≤5)	3
Rotatable bonds	5
Polar surface area (131.6 Å^2^)	96.22 Å^2^

**Table 3:** In silico ADMET analysis of α-mangostin.

Parameters	Results
ADMET solubility level ^a^	1
ADMET absorption level ^b^	2
CYP2D6	Non-inhibitor
Hepatotoxicity	Toxic
ADMET BBB level ^c^	4
ADMET PPB ^d^	True

^a^ ADMET solubility level: 0 (extremely low), 1 (no, very low, but possible), 2 (yes, low), 3 (yes, good), 4 (yes, optimal), 5 (no, too soluble), 6 (unknown). ^b^ ADMET absorption level: 0 (good), 1 (moderate), 2 (poor), 3 (very poor). ^c^ ADMET BBB (blood–brain barrier) level: 0 (very high), 1 (high), 2 (medium), 3 (low), 4 (undefined). ^d^ ADMET PPB (plasma protein binding): True—highly bound, more than 90%; False—poorly bound, less than 90%.

**Table 4:** TOPKAT analysis of α-mangostin.

Parameters	Results
Aerobic biodegradability	Non-degradable
Ames mutagenicity	Non-mutagen
Ocular irritation	Mild irritant
Skin irritation	Non-irritant
Rat female NTP	Non-carcinogen
Rat male NTP	Carcinogen
Rat oral LD_50_ (g/kg)	0.168
Rodent carcinogenicity	Non-carcinogen

**Table 5:** Binding free energy of α-mangostin.

Contribution Energy	kcal/mol
Electrostatics energy (ΔE_electrostatics_)	−6.13
Van der Waals (ΔE_vdW_)	−51.5655
Polar solvation energy (ΔG_PB_)	46.8995
Non-polar solvation (ΔG_SA_)	−5.4695
Gas phase molecular mechanics (ΔE_gas_)	−57.6956
Solvation free energy (ΔG_sol_)	41.43
Polar binding free energy (ΔG_polar_)	40.7695
Non-polar binding free energy (ΔG_non-polar_)	−57.0351
Free binding energy (ΔG_bind_)	−16.2656

## Data Availability

Data are contained within the article.
